# From tumor‐centric to ecosystem‐based hypotheses in brain tumor research and care: Proceedings from The second Brain Tumor Meeting by the Sea organized by Julie Gavard and Eric Chevet, held in St Malo (France), 23–25 March 2026

**DOI:** 10.1002/1878-0261.70298

**Published:** 2026-07-04

**Authors:** Julie Gavard, Flavie Bénard, Eva Billat, Margaux Le Guyon, Victoria Maltret, Laura Merlet, Chloé Astrid Walter, Tony Avril, Eric Chevet

**Affiliations:** ^1^ CRCINA, CNRS, Inserm Nantes Université, Université Angers France; ^2^ Équipe Labellisée Ligue contre le Cancer Paris France; ^3^ Institut de Cancérologie de l'Ouest Saint Herblain France; ^4^ UMR1242 INSERM University of Rennes France; ^5^ Équipe Labellisée Fondation pour la Recherche Médicale Rennes France; ^6^ Comprehensive Cancer Center Eugène Marquis Rennes France

**Keywords:** adult brain tumors, microenvironment, pediatric brain tumors, plasticity, stress

## Abstract

Brain tumors remain among the most lethal cancers, largely due to their remarkable heterogeneity, plasticity, and resistance to therapy. The second Brain Tumor Meeting by the Sea (Saint‐Malo, France, 2026) brought together researchers, clinicians, and patient representatives to discuss emerging concepts shaping the future of neuro‐oncology. A recurring theme was the shift from a tumor‐centric perspective toward an ecosystem‐based view that integrates tumor cells, microenvironmental cues, developmental context, and patient‐centered dimensions. Advances in patient‐derived models, multi‐omics approaches, spatial technologies, and artificial intelligence are refining tumor classification and revealing novel therapeutic vulnerabilities. Discussions highlighted cellular plasticity and stress‐adaptation mechanisms as key drivers of tumor evolution and treatment resistance. They also emphasized the need for identifying dynamic biomarkers and developing more physiologically relevant disease models. Beyond biological discoveries, the meeting underscored the importance of strengthening interactions among research, clinical care, and patient communities. Together, these advances support a more integrated framework for understanding brain tumors and developing future therapeutic strategies.

## Graphical abstract

Primary brain tumors, whether in adults or children, present a major challenge because of their dramatic prognosis and the ongoing lack of efficient therapeutic approaches. In recent years, a shift has occurred from tumor‐centric concepts to a more holistic view of these tumors as dynamic ecosystems. Here, we discuss how this change may impact patient care and recovery, as explored during the ‘Second Brain Tumor Meeting by the Sea’.

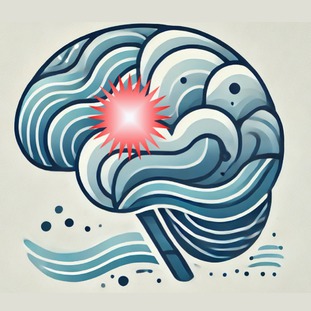

**Abbreviations**
BTMBTS: brain tumor meeting by the sea
CAR‐T cell: chimeric antigen receptor T cell
ecDNA: extrachromosomal DNA
EVs: extracellular vesicles
GB or GBM: glioblastoma
GOPA: *GrOupe* Préclinique de l'ANOCEF, l'Association des Neuro‐Oncologues d'Expression Française
HDAC3: Histone deacetylase 3
HGGs: high‐grade gliomas
IAPs: inhibitors of apoptosis proteins
IDH: isocitrate déshydrogénases
IRE1: inositol requiring enzyme 1
LIPU: low‐intensity pulsed ultrasound
MES: mesenchymal
MOFA: multi‐omics factor analysis
PARP: poly(ADP‐ribose)‐polymerase
PDO/X: patient‐derived organoids and xenografts
PETRA: Réseau PrEclinique et TRAnslationnel de recherche en neuro‐oncologie
pHGG: Pediatric high‐grade gliomas
WT: wild‐type

## 1 Introduction: Why do brain tumors remain uniquely challenging?

High‐grade brain tumors are from different cellular origins and etiologies depending on their pediatric or adult context. Glioblastoma (GB or GBM) is the most common malignant primary brain tumor in adults, representing approximately 80% of lethal primary brain cancers in the United States, with an incidence of about 3/100.000 [1]. The prognosis for GB remains poor with a median survival ranging between 15 and 16 months postdiagnosis and an average age at diagnosis of about 50 years [2]. Only approximately 5% of patients survive more than 5 years [3]. GB is characterized by a high proliferation rate, core necrosis, microvascular proliferation, and tumor infiltration in the surrounding brain parenchyma. The standard of care in GB consists of surgical tumor resection and radiotherapy plus concomitant chemotherapy with temozolomide [4]. Resistance to treatment is largely driven by residual tumor cells that escape tumor resection and invade the normal brain parenchyma, as well as the extreme plasticity of the remaining GB cells. Recent studies illustrated that GB is composed of switchable cellular states that dynamically respond to their environments, therefore contributing to cellular adaptation and therapeutic resistance. Recent developments in immunotherapy did not succeed in overcoming this challenge.

Pediatric high‐grade gliomas (pHGG) form a heterogeneous group of tumors classified into three main molecular types: H3K27‐altered diffuse midline glioma, H3G34‐mutant hemispheric glioma, and H3‐wild‐type/IDH‐wild‐type (IDH‐WT) tumors [5]. Regarding their therapeutic handling, surgical resection remains a key option when possible. It can be followed by focal radiotherapy and concomitant chemotherapy. Standard chemotherapies include temozolomide, although pediatric tumors exhibit distinct biology compared with adult forms, thereby limiting extrapolation of adult protocols [6]. Emerging diagnostic approaches to refine tumor stratifications are now guiding trial eligibility for novel therapeutic strategies. Together and for both adult and pediatric tumors, these approaches represent a global trend to advance patient care from tumor‐centric and histology‐based treatments to precision medicine. They are taking into consideration factors expanding beyond current dogmas to the notion of tumor ecosystems that remain to be fully characterized. This concept stemmed from the presentations and discussions that occurred in the second French Brain Tumor Meeting By The Sea (BTMBTS) in St‐Malo, France in March 2026, under the auspices of GOPA/PETRA networks (*GrOupe* Préclinique de l'ANOCEF, l'Association des Neuro‐Oncologues d'Expression Française, and Réseau PrEclinique et TRAnslationnel de recherche en neuro‐oncologie).

## 2 Multidimensional data integration and machine learning to better define tumors

In order to facilitate the transition toward precision approaches in neuro‐oncology, several teams have initiated strategies relying on patient‐derived organoids and xenografts models (PDO/X) to not only characterize the tumors at both cellular and molecular levels but also to provide a unique drug screening framework. This was illustrated by Dr Anna GOLEBIEWSKA (NORLUX, Luxembourg) who presented an integrative approach combining high‐throughput drug screening with multi‐omics factor analysis (MOFA) in PDOs to classify high‐grade gliomas (HGGs) subtypes and identify potential actionable vulnerabilities. This led to the identification of IDH‐mutant high‐grade gliomas (HGGs) dependency on HDAC3, a feature not found in IDH‐WT tumors, as well as the identification and refinement of a specific rare tumor subgroup encompassing MYCN amplification. Even though this approach resulted in significant enhancement of knowledge about HGGs, several biases remain such as the discrimination between intrinsic and acquired resistance in PDO/X thereby challenging the discovery of new drugs applicable to the clinic [7, 8]. This led to the identification and refinement of a specific rare tumor subgroup with genetic alterations. In addition to the use of tumor‐derived cellular tools, another approach consists of using large‐scale analyses of tumor characteristics to predict patient survival. In this context, an approach using machine learning models applied to histological slides from a cohort of 548 GB IDH‐WT patients enriched in long survivors and combined with spatial transcriptomic analyses aimed at finding prognostic markers and predicting survival. The model successfully stratified tumors according to short‐ or long‐term survival and discriminated tumors bearing FGFR3::TACC3 fusions from other GB. Furthermore, cell‐type deconvolution based on spatial transcriptomic information revealed that tumors enriched in cells exhibiting a mesenchymal (MES)‐like state at the invasive margin were associated with shorter patient survival [9]. Collectively, the results presented in this session not only demonstrate the relevance of multiscale approaches to better characterize brain tumors but also point toward the relevance of the information collected in the clinical context.

**Take‐Home Message**. Brain tumor classification is evolving from the analysis of individual molecular alterations toward the integration of multiscale datasets encompassing genomic, spatial, and functional information. These studies that combine refined models and multi‐omics datasets assisted with machine learning highlight the potential of data integration to improve patient stratification and advance precision oncology.

## 3 Plasticity as a central mechanism for resistance

**Fig. 1:**
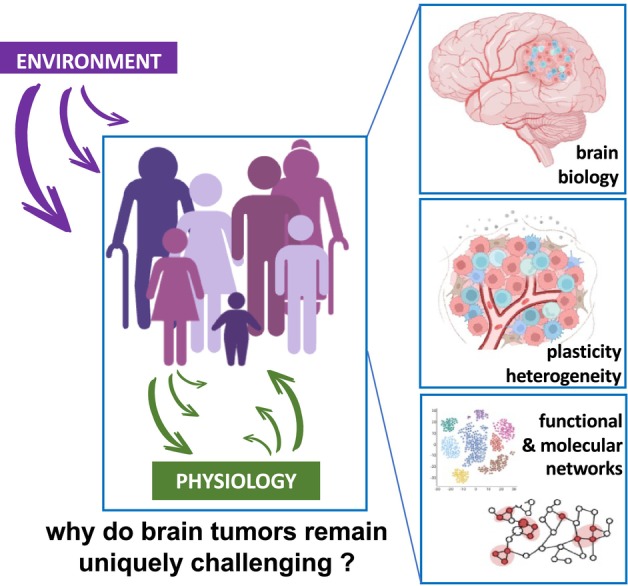
Overview of the tumor ecosystem‐driven framework applied to primary brain tumors in adults and children. The exploration level goes from population studies (including exposure to challenges from the environment and physiological specificities) to brain biology, and cellular and molecular characterization.

A major theme spanning most sessions was the role of cellular plasticity as a driver of therapeutic resistance in brain tumors. Rather than representing static diversity, tumor heterogeneity was discussed as a dynamic and adaptive process enabling tumor cells to survive under versatile pressure. This view is increasingly supported with the democratized use of single‐cell transcriptomics, spatial profiling, and lineage tracing, showing that glioma cells can reversibly transition between states in response to environmental stress and therapy (Fig. 1). In this line, we collectively question how lineage switching contributes to resistance mechanisms, as tumor cells are able to transition between cellular identities in response to environmental and treatment‐related constraints. These concepts resonate with oral and poster presentations suggesting that tumor cells hijack developmental programs and oscillate between neural progenitor‐like, oligodendroglial‐like, and mesenchymal‐like states rather than remaining locked into a single lineage identity. Furthermore, the lecture from Benjamin WERNER (Barts Cancer Institute, London, UK) highlighted the role of extrachromosomal DNA (ecDNA) as a potential driver of tumor plasticity and evolutionary adaptation [10]. ecDNA are circular DNA fragments ranging from approximately 0.5 to 5 Mbp that arise from chromosomal instability and frequently harbor oncogenes, such as EGFR. Although detected in approximately 20–25% of cancers, their abundance varies substantially between tumor types and is consistently associated with poor prognosis. A stochastic mathematical model accurately reproduced the heterogeneous distribution of ecDNA during cell division [11]. Despite their uneven segregation, ecDNA persist over time within tumor populations, suggesting that they confer a selective evolutionary advantage that remains incompletely understood. Supporting this hypothesis, genetically engineered mouse neural stem cells carrying ecDNA‐mediated MYC amplification maintained these structures for several months *in vivo* as well as in derived cultures *in vitro*. Together, these findings position ecDNA as both a source of genetic heterogeneity and a dynamic mechanism supporting adaptive cellular states and tumor evolution.

Additional studies support the central role of stemness in glioblastoma resistance and adaptation. For instance, molecules initially associated with cell survival signaling, including inhibitors of apoptosis proteins (IAPs), also contribute to maintaining glioma cells in a stem‐like state [12]. Inhibition of these pathways promotes cellular differentiation and increases treatment sensitivity, suggesting that stemness is not a fixed property but a reversible and therapeutically targetable state. Beyond tumor cell autonomous mechanisms, other presentations highlight the importance of the microenvironment in sustaining plasticity, notably through inflammatory and immunosuppressive signaling, reinforcing the concept that stemness emerges from dynamic interactions between tumor‐intrinsic programs and the surrounding niches. Likewise, treatment‐induced plasticity was discussed as a direct consequence of therapeutic pressure. Treatments themselves may reshape tumor cell identity, differentiation status, and behavior, thus selecting for phenotypes that promote survival, invasion, and recurrence [13]. Indeed, chemo‐ and radio‐therapies may not only select resistant clones but also induce adaptive cellular reprogramming that contributes to therapeutic resistance [14].

**Take‐Home Message**. Tumor heterogeneity should not be viewed merely as pre‐existing diversity, but as a dynamic process of adaptation. Cellular plasticity emerges as a central mechanism through which brain tumors evolve, resist treatment, and eventually recur. The convergence of developmental biology, single‐cell technologies, and evolutionary models is progressively redefining resistance as an active and reversible process of state transition rather than a consequence of clonal selection alone.

## 4 Stress adaptation as therapeutic opportunities

Multiple presentations underscored the importance of cellular stress adaptation in shaping brain tumor evolution (Fig. 1). While historically viewed as mechanisms that enable cancer cells to survive hostile microenvironments and therapeutic insults, growing evidence suggests that these adaptive stress‐based programs also create exploitable vulnerabilities. This conceptual shift is particularly relevant to brain tumors, where genetic drivers, metabolic constraints, and microenvironmental pressures converge to shape highly plastic cellular states. Recent work highlights how oncogenic alterations can directly rewire stress‐adaptation pathways. Mutations in IDH, for example, not only alter cellular metabolism through the accumulation of the oncometabolite D‐2‐hydroxyglutarate but also reshape epigenetic and transcriptomic landscapes [15]. Likewise, studies of telomere maintenance mechanisms continue to reveal previously unrecognized glioma subtypes, emphasizing how adaptation to replicative stress contributes to tumor diversification and may challenge current classification frameworks [16]. Beyond genetic alterations, metabolic heterogeneity emerged as a major determinant of tumor behavior [17]. Distinct GB cellular states display differential dependencies on glucose or glutamine metabolism, and nutrient deprivation can actively drive transitions toward more aggressive mesenchymal phenotypes. These observations support a model in which metabolic stress is not simply tolerated by tumor cells but actively fuels cell plasticity and disease progression through interactions with the tumor microenvironment [18, 19].

At the same time, increasing attention was paid to the intracellular systems that allow cancer cells to cope with proteotoxic and environmental stress. The unfolded protein response regulator IRE1 exemplifies this complexity, orchestrating transcriptional and post‐transcriptional programs that influence not only tumor cell fitness, but also angiogenesis, invasion, and immune‐cell recruitment [20, 21]. Similarly, emerging studies of autophagy‐related pathways, endosomal trafficking, and vesicle dynamics suggest that mechanisms controlling intracellular organization are intimately linked to GB invasion and adaptation [22, 23, 24]. The identification of novel regulators of endolysosomal homeostasis further expands the spectrum of stress‐response pathways that may be therapeutically targeted [25]. Likewise, large‐scale combination screens have highlighted the potential of simultaneously targeting multiple core vulnerabilities, revealing synergistic therapeutic interactions that may enhance treatment efficacy [26].

**Take‐Home Message**. Rather than representing merely a hallmark of cancer cell resilience, stress adaptation appears to function as a central organizing principle linking metabolism, cellular plasticity, tumor–microenvironment interactions, and invasion. Understanding how brain tumors balance these adaptive responses may reveal novel therapeutic opportunities capable of transforming mechanisms of survival into liabilities that can be selectively targeted.

## 5 Translational bottlenecks: From biological insight to patient benefit

Despite these advances, translating mechanistic insights into meaningful clinical benefit remains a major challenge. Several presentations highlighted complementary aspects of this translational gap, spanning disease modeling, biomarker development, and therapeutic intervention. A recurring theme was the need for experimental models that better capture the developmental and microenvironmental brain tumor contexts [27]. In this regard, both short talks and poster presentations highlighted efforts to improve preclinical models, ranging from primary tumor cells, glioma stem cell cultures, and spheroids to more sophisticated approaches, such as induced pluripotent stem cell‐derived brain organoids. These models revealed variant‐specific effects on neurodevelopment, genomic stability, and tissue organization, illustrating how organoid systems can provide access to the earliest stages of tumor predisposition that remain inaccessible in conventional models. More importantly, the lecture from David CASTEL (IGR, Villejuif, France) emphasized that pediatric tumor initiation and therapeutic response cannot be fully understood through genetics alone [28]. Diffuse midline gliomas arise from a limited number of genetic alterations but display a strong dependence on developmental state, anatomical location, and epigenetic context. These observations challenge reductionist genetic models and support the development of experimental systems capable of recapitulating cellular identity and developmental trajectories.

The translation of biological discoveries into therapeutic strategies was addressed from several complementary perspectives, reviewing ongoing efforts to improve outcomes beyond the standard Stupp regimen. This includes PARP inhibition, immune checkpoint blockade, dendritic cell vaccines, CAR T‐cell therapies, and drug repurposing approaches [29]. However, the delivery of effective therapies to brain tumors remains a critical obstacle. For instance, emphasis was made around low‐intensity pulsed ultrasound (LIPU), a strategy designed to transiently open the blood–brain barrier and enhance drug penetration into the tumor [30]. Beyond its effects on drug delivery, LIPU appeared capable of modulating the tumor microenvironment, highlighting the complexity of translating technological innovations into predictable biological outcomes. Another challenge concerns the identification of biomarkers capable of capturing tumor dynamics in real time. Extracellular vesicle (EVs) might help fill this gap. These membrane‐enclosed nanoparticles, which selectively package and transport cellular components into the extracellular space, could serve as accessible biomarkers of tumor state and microenvironment adaptation [31]. They may also provide valuable insights into the mechanisms of intercellular communication within gliomas.

**Take‐Home Message**. It has to be emphasized that overcoming translational bottlenecks will require more than the identification of new molecular targets. Progress will depend on the development of physiologically relevant models, robust biomarkers of tumor evolution, and therapeutic strategies capable of addressing both the biological and physical barriers that limit treatment efficacy in gliomas.

## 6 Creating a multidimensional ecosystem for neuro‐oncology

**Fig. 2:**
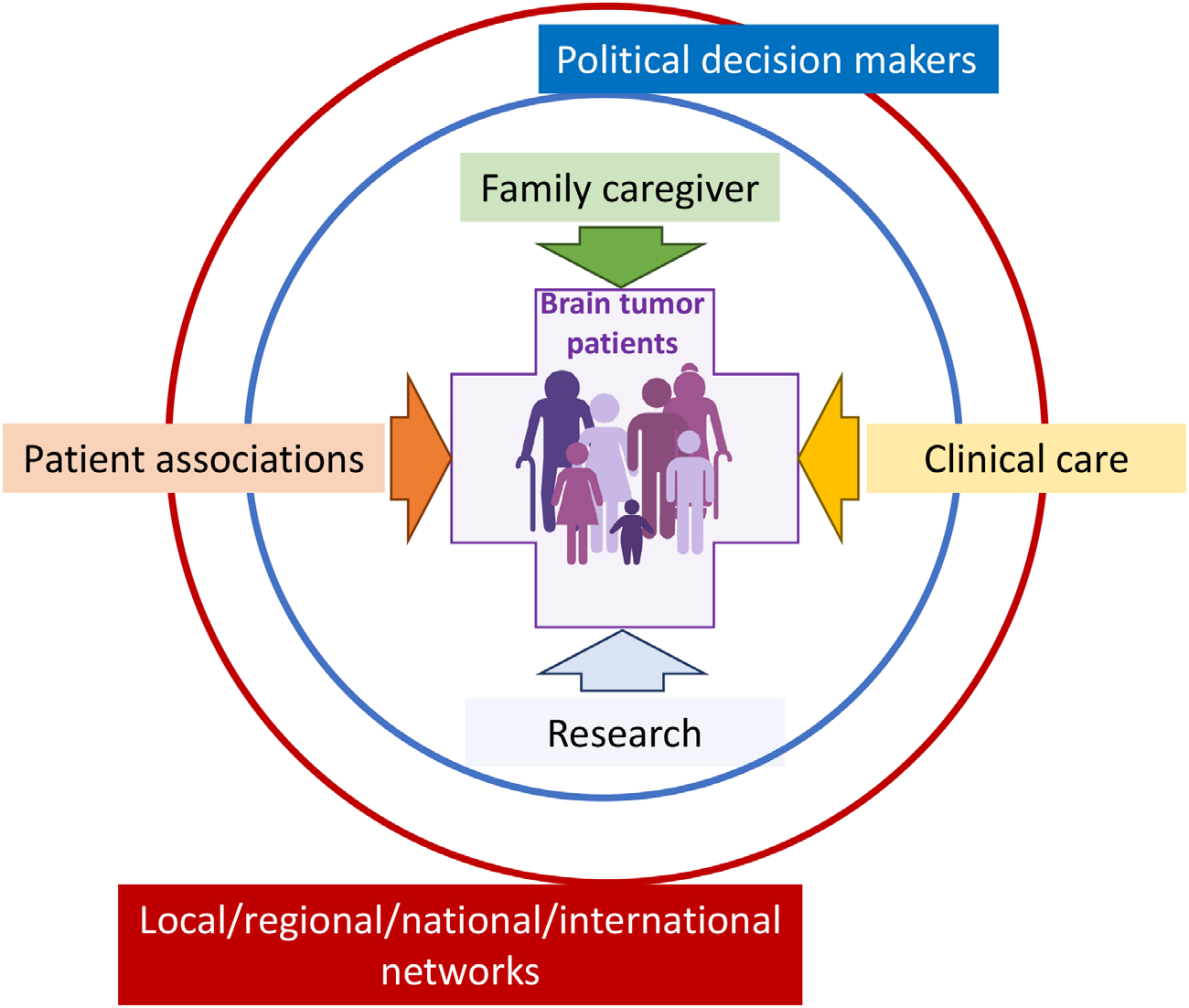
Extended neuro‐oncology ecosystem integrating research (light blue), clinical care (yellow), patient associations (orange), family caregivers (green), regional and national networks (red), and policy makers (dark blue).

To improve patient care, successful initiatives have previously proposed to integrate additional dimensions to patient care, such as research and/or patient experts (Fig. 2). This is exemplified through international schemes, such as the International Brain Tumor Alliance (IBTA, https://theibta.org/). One opportunity could be represented by initiating regional/national multipartite ecosystems for patients suffering from brain tumors: connecting research, care (neurosurgery, radiology, oncology, and supportive care), patient communities, including patient associations and beyond. The interest of such an endeavor was discussed at BTMBTS26, bringing together translational researchers, medical‐care professionals, patients, and a brain tumor patient association (Oligocyte Bretagne, https://oligocyte‐bretagne.fr). While in general each association has their own expectations and aims, several common expectations have emerged. These include regular communication from researchers, through short presentations, patient meetings, laboratory visits, or annual progress reports. They could as well involve sustained interactions with healthcare professionals to support patients and caregivers throughout their care journey.

Reciprocally, for researchers and medical‐care professionals, patient associations are a reminder of the relevance of their work. This is particularly valuable for basic and translational researchers whose activities may remain distant from direct patient interactions. Currently, the neuro‐oncology field remains fragmented, with research, clinical practice, and patient communities frequently operating in silos. Consequently, creating dedicated spaces for dialogue and the sharing of successes, challenges, and struggles (by fully integrating patient perspectives) should become a priority. Such initiatives would contribute to developing an expanded, multiscale ecosystem capable of accelerating progress to fight against brain tumors.

## 7 Concluding on future priorities in brain tumor research

The presentations and discussions held during the 2^nd^ BTMBTS highlighted the extent to which neuro‐oncology is shifting from tumor‐centric models to ecosystem‐based approaches to better understand and treat brain tumors. This evolution encompasses not only the development of an expanding panel of precision therapeutics but also the identification of more informative and dynamic biomarkers, increasingly refined patient stratification schemes as well as adaptive clinical trial designs. Together, these advances provide a framework that the brain tumor community must now translate into routine research and clinical practice. Strengthening interactions between adult and pediatric neuro‐oncology communities will all be essential to facilitate knowledge transfer and identify shared biological concepts whenever possible. Furthermore, the tumor ecosystem concept may provide a useful framework for reconsidering how brain metastases, those secondary tumors developing into the brain, are studied and managed, extending these ideas beyond primary brain tumors.

Beyond research and clinical care, the development of integrated networks involving patients' associations, patient experts, and even family caregivers could substantially enhance patient support and engagement at locally, regional, national and international levels. Such networks would not only provide the critical mass required to support public awareness campaigns (e.g. Brain Tumor Awareness Month, May in Gray) but could also strengthen interactions with policy makers and funding agencies, to inform them on the considerable challenges posed by brain tumors (Fig. 2).

## Conflict of interest

EC is the founder of Thabor Therapeutics and Exa Noma Therapeutics AB. The other authors declare no conflict of interest.

## Author contributions

JG and EC conceived, redacted the manuscript, and prepared the figures. TA corrected the first draft of the manuscript. FB, EB, MLG, VM, LM, and CAW took notes during the presentations. All the authors read the final draft of the manuscript.
